# Iron carbide nanoplatelets: colloidal synthesis and characterization†

**DOI:** 10.1039/c9na00526a

**Published:** 2019-10-07

**Authors:** Frank M. Abel, Shirin Pourmiri, Georgia Basina, Vasileios Tzitzios, Eamonn Devlin, George C. Hadjipanayis

**Affiliations:** Department of Physics and Astronomy, University of Delaware Newark DE 19716 USA fabel@udel.edu frank.m.abeliii@gmail.com; Department of Chemical Engineering, Khalifa University of Science and Technology, Petroleum Institute P. O. Box 2533 Abu Dhabi United Arab Emirates; Institute of Nanoscience and Nanotechnology, NCSR Demokritos Athens 15310 Greece v.tzitzios@inn.demokritos.gr

## Abstract

Iron carbide nanoplatelets with an orthorhombic Fe_3_C structure were synthesized following a simple liquid chemical approach. The formation of the carbide phases was shown to depend on the presence of a long chain diol and the reaction temperature. Confirmation of the iron carbide phases and structural characterization was made by X-ray diffraction (XRD) and Mössbauer spectroscopy. Particle morphology was characterized by transmission electron microscopy (TEM) and HR-TEM and the magnetic properties were measured with magnetometry (VSM). The sample with the Fe_3_C phase shows a ferromagnetic behavior with a magnetization of 139 emu g^−1^ under a 30 kOe applied field. The simple methodology presented here for producing iron carbide nanoplatelets has promising application in the biomedical and catalyst industries.

## Abstract 2

Single-step liquid chemical synthesis of iron carbide nanoplatelets.

## Graphical abstract

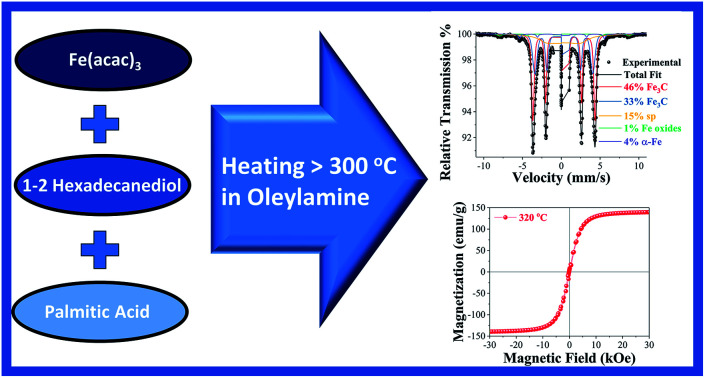

## Introduction

Nanostructured magnetic materials exhibit unique and interesting properties from scientific and technological perspectives. Since World War II, metal carbides have attracted great interest for the development of high stress-resistance materials. Because of the electron-rich metal–carbon bonds, metal carbides display novel magnetic, catalytic, and electronic properties.^1^

The development of simple synthesis methods is critical for the implementation of magnetic nanomaterials in future technologies such as biomedicine, permanent magnets, and catalysts.^2^ Iron carbide nanoparticles have potential applications in biomedicine as MRI contrast agents and in hyperthermia cancer therapy due to their high magnetic moment and magnetic and chemical stability.^3,4^ The catalytic properties of iron carbides are also of interest in the Fischer–Tropsch process which is used for producing liquid fuels from a variety of sources.^4,5^ The limiting factor in utilizing iron carbide nanomaterials for these applications is the lack of simple methods for producing high quality single phase materials and the ability to tailor their size and shape for a given application. There exist numerous methods for producing iron carbide phases, such as high pressure and temperature methods,^6–8^ laser^9–12^ and physical^13–16^ pyrolysis, sol–gel synthesis,^17–20^ gas–solid methods,^21–23^ sonolysis followed by thermal treatment,^24^ flame spray synthesis,^25^ laser ablation,^26^ and thermal treatment of amorphous oxides.^27^ However, these methods typically lead to mixtures of various iron carbide phases and poor nanostructural uniformity.^3,4^ More recently, liquid phase chemical methods have been found to produce uniform iron carbide nanoparticles; however, the methods usually use multistep processes and harsh precursors such as Fe(CO)_5_.^3,4^ Additionally, Ge *et al.* showed that iron carbide nanoparticles could be synthesized by using amorphous Fe nanoparticles produced by sodium borohydride reduction of iron(ii) chloride, which were then heated in oleylamine to 330 °C for different times producing the Fe_5_C_2_ structure.^28^

In this study, we report a colloidal synthesis of iron carbide nano-platelets. Chemical synthesis leads to single iron carbide phase materials with 79% Fe_3_C with a magnetization of 139 emu g^−1^ under a 30 kOe applied field. Identification of the Fe_3_C phase and phase percentage is carried out by Mössbauer spectroscopy. From our knowledge, this is the first single-step liquid chemical synthesis of Fe_3_C nanoplatelets produced without the use of an Fe(CO)_5_ iron precursor.

## Experimental methods

### Synthesis of iron carbide

The samples were synthesized by combining Fe(acac)_3_, 1,2-hexadecanediol, and palmitic acid as the surfactant in oleylamine as the primary solvent at room temperature. The precursors were magnetically stirred for 2 hours under continuous purging with H_2_/Ar gas. A typical experiment used 0.5 mmol Fe(acac)_3_, 2 mmol 1,2-hexadecanediol, and 200 mg palmitic acid in 20 mL oleylamine. To produce a higher yield for Mössbauer spectroscopy measurements, the precursor quantities were quadrupled in the same solvent volume for the two experiments at the highest reaction temperatures. The solution was then heated to temperatures between 260 °C and 320 °C for 1 hour. An additional experiment was performed in which the solution was heated to 300 °C for 1 hour followed by heating to 340 °C for 2 hours. After cooling the solution to room temperature, the material was precipitated with ethanol and magnetically separated from the solution. Additional washing was done using ultra-sonication and magnetic separation with both ethanol and hexane to remove excess organic material. The materials were either dried in air/desiccator, forming a black powder, or dispersed in ethanol for characterization.

### Materials characterization

The crystal structure of the materials was determined using X-ray diffraction (XRD, Rigaku Ultima IV) with Cu Kα radiation; additional structural characterization and phase analysis were performed by Mössbauer spectroscopy (constant acceleration spectrometer, Janis cryostat, Co^57^(Rh) source, calibrated with thin α-Fe foil) on selected samples. The size and morphology of the particles were determined using transmission electron microscopy (TEM, JEOL JEM-3010) and the magnetic properties were measured with a 3 Tesla vibration sample magnetometer (VSM, Quantum Design).

## Results and discussion

**Fig. 1:**
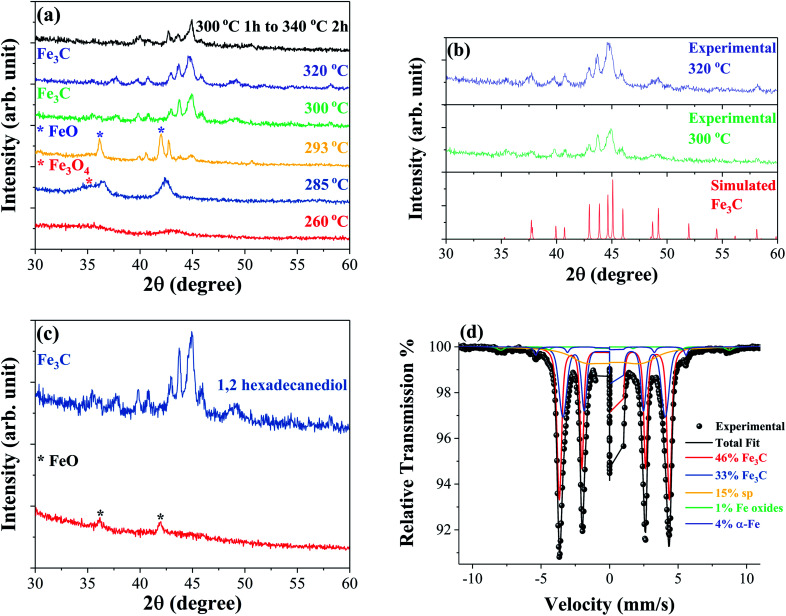
XRD patterns measured at room temperature of samples synthesized at different reaction temperatures (a), samples synthesized at 300 °C and 320 °C in comparison to the simulated Fe_3_C structure obtained using PowderCell.^29^ Structural data for orthorhombic Fe_3_C simulations acquired from ICSD-99002 (b) and samples synthesized at 300 °C for 1 hour with and without 2 mmol 1,2-hexadecanediol (c). Mössbauer spectroscopy performed at 80 K of a sample synthesized at 320 °C confirming the formation of the Fe_3_C structure (d).

The formation mechanism and crystal structure of iron carbide were studied using powder X-ray diffraction (XRD). Fig. 1(a) shows the XRD patterns of samples synthesized at different reaction temperatures. The data show that identifiable iron carbide structures begin to form at 300 °C. The XRD patterns of samples synthesized at 300 °C and 320 °C are compared to simulated reference patterns in Fig. 1(b), and show the main phase to be orthorhombic Fe_3_C. The step reaction performed at 300 °C for 1 hour followed by 340 °C for 2 hours leads to a mixture of Fe–C phases. At 260 °C, two broad peaks are observed indicating an amorphous or nanocrystalline material. The main peak observed at 42.98° has a corresponding *d*-spacing of 0.210 nm; the peaks and relative intensities have similarities to FeO peaks, but are shifted to higher angles possibly indicating that under these conditions, an iron rich FeO structure forms. Increasing the temperature to 285 °C causes the main peak of the structure to shift to a lower angle of 42.42°, corresponding to a *d*-spacing of 0.213 nm, possibly indicating a similar iron rich FeO structure. The shoulder to the left of the peak at ∼36° is believed to belong to either Fe_3_O_4_ or Fe_2_O_3_. At 293° the main phase is clearly identified as FeO with a main peak at 41.99°, corresponding to a *d*-spacing of 0.215 nm, coinciding with the (200) diffraction plane. In addition to the FeO phase, there are one or more unidentifiable phases which may be intermediate Fe–C structures. Fig. 1(c) compares a reaction performed at 300 °C as described in the experimental section but without the addition of the 1,2-hexadecanediol reducing agent. The XRD patterns show that in the absence of 1,2-hexadecanediol only the FeO phase, identified by the peaks at ∼42° and ∼36°, forms. With the addition of 1,2-hexadecanediol, the iron carbide phase Fe_3_C forms. The results suggest clearly that the carbon source for metal carbide formation is the 1,2-hexadecanediol molecule. The carbon becomes available *via* the oxidation cleavage of the diol into esters, ketones or aldehydes in the presence of Fe^3+^ ions acting as the oxidant, which are then reduced to the Fe^2+^ state.

Mössbauer spectroscopy was performed on selected samples to identify the iron carbide phases formed during the synthesis. Fig. 1(d) shows the Mössbauer spectrum of the sample synthesized at 320 °C obtained at 80 K; the fitting analysis confirms Fe_3_C as the majority phase, making up 79% of the sample. Two subspectra are used to approximate the Fe_3_C (46% and 33%) phase indicating some slight compositional variation in the phase. In addition to Fe_3_C, the spectra reveal small percentages of ferromagnetic iron oxides, α-Fe, and a relaxing/superparamagnetic (sp) component. Mössbauer spectroscopy was also performed on the sample produced by the step reaction with a synthesis temperature of 300 °C for 1 hour followed by 340 °C for 2 hours. The spectrum obtained at 4.2 K reveals a mixture of Fe_5_C_2_, Fe_3_C, and iron oxide phases in this sample, as shown in the ESI Fig. S1.†

**Fig. 2:**
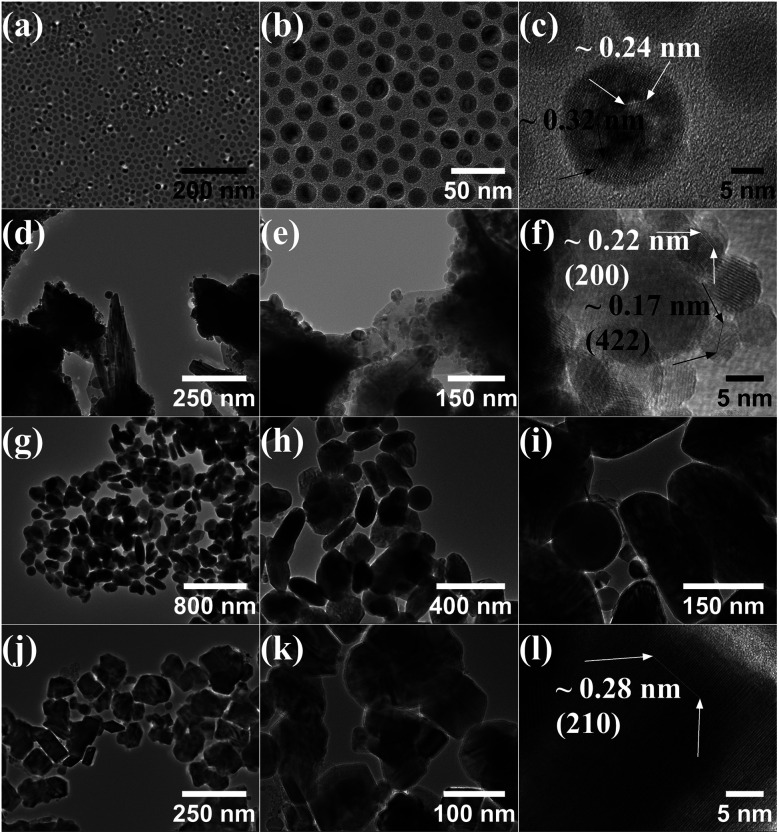
TEM of samples synthesized at 285 °C (a–c), 300 °C (d–f), 320 °C (g–i), and 300 °C for 1 hour followed by 340 °C for 2 hours (j–l).

The size and morphology of the particles obtained by TEM for the samples synthesized at 285 °C, 300 °C, 320 °C, and step reaction with maximum temperature of 340 °C are shown in Fig. 2(a–l). At 285 °C the particles show a uniform spherical morphology, Fig. 2(a and b). HR-TEM in Fig. 2(c) suggests a possible core–shell or polycrystalline structure with a different *d*-spacing for the surface *versus* the interior of the particle. When the reaction temperature is increased to 300 °C, corresponding to the emergence of the Fe_3_C phase, the morphology changes significantly showing variable sized spherical particles together with larger platelet structures, Fig. 2(d and e). The smaller spherical particles appear on the surface or within the platelet structures. HR-TEM in Fig. 2(f) shows the *d*-spacing measurements of two of the spherical particles with a *d*-spacing of 0.17 nm, close to that of the Fe_3_O_4_ diffraction plane (422), and a *d*-spacing of 0.22 nm, close to that of the FeO diffraction plane (200). At 320 °C the material shows a more uniform platelet morphology with an average size of 210 nm found by averaging the approximate length and width of a selection of particles from TEM micrographs. Small spherical nanoparticles are no longer clearly observed on the surface/interior of the platelets, Fig. 2(h and i). In the step reaction, more facetted platelets are observed compared to the material synthesized at 320 °C with HR-TEM *d*-spacing measurements giving a value of 0.28 nm, close to that of the Fe_5_C_2_ (211) diffraction plane.

**Fig. 3:**
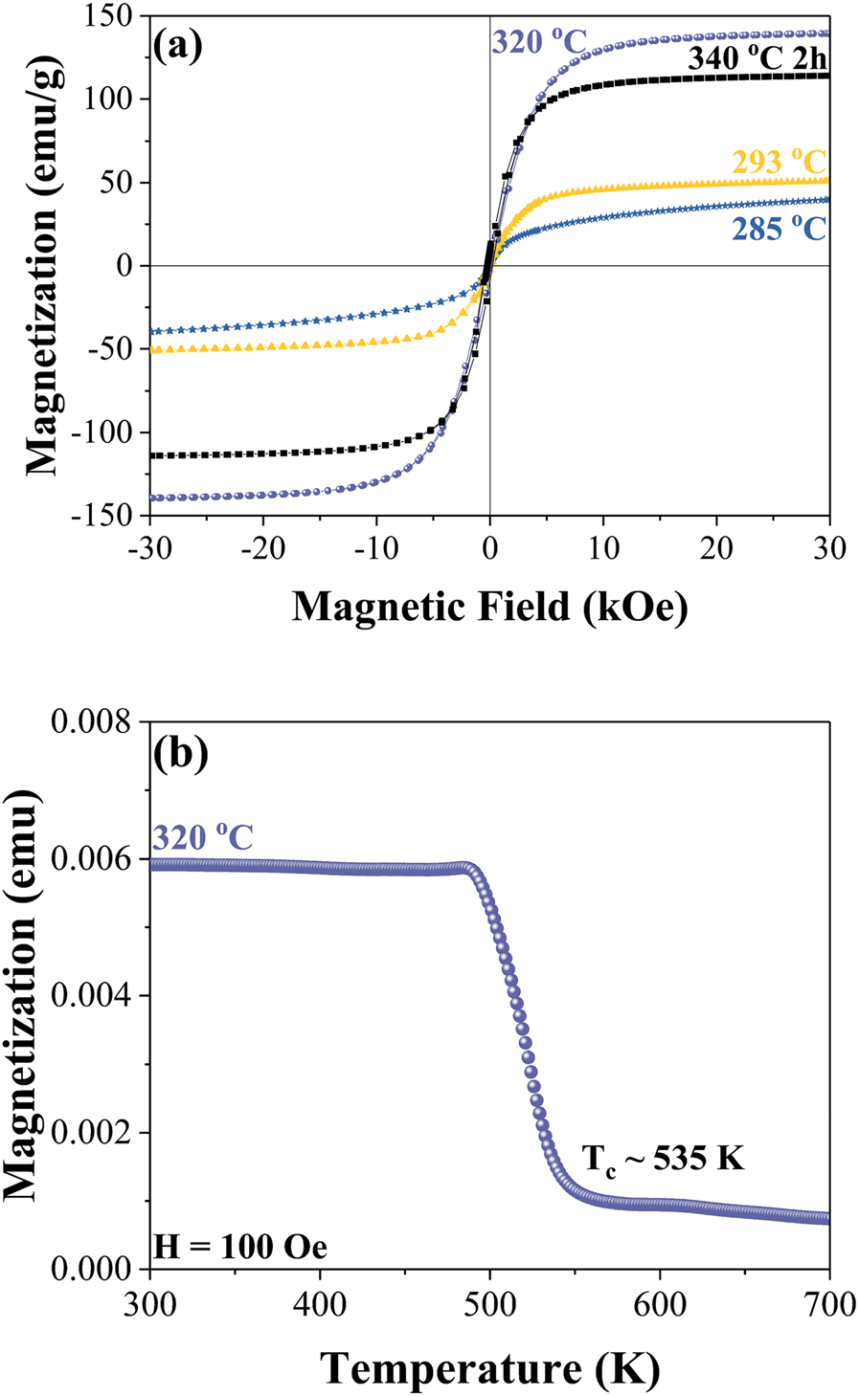
Room temperature hysteresis curves of samples synthesized at 285 °C (blue), 293 °C (yellow), 320 °C (purple), and 300 °C for 1 hour followed by 340 °C for 2 hours (black) (a). Thermomagnetic measurements of the sample synthesized at 320 °C showing an approximate Curie temperature of 535 K (b).

Room temperature hysteresis loops are shown in Fig. 3(a) for the samples synthesized at 285 °C, 293 °C, 320 °C, and after a step reaction with a maximum temperature of 340 °C. At 285 °C the nanoparticles are most likely superparamagnetic at room temperature with zero coercivity; increasing the synthesis temperature to 293 °C results in observable ferromagnetic behaviour with a coercivity of 141 Oe and a magnetization of 51 emu g^−1^ under a 30 kOe applied field. When the Fe_3_C phase is formed, the magnetization increases to 139 emu g^−1^ (at 30 kOe) with a coercivity of 165 Oe. In the step reaction sample, which leads to a mixture of Fe_3_C and Fe_5_C_2_ phases, the magnetization at 30 kOe decreases to 114 emu g^−1^ with a coercivity of 226 Oe. The decrease in magnetization relative to the sample with the majority Fe_3_C nanoplatelets is most likely due to the increased carbon content in the Fe_5_C_2_ phase, in addition to slight differences in iron oxide phases and α-Fe. Thermomagnetic measurements were performed on the sample with the majority Fe_3_C phase synthesized at 320 °C, as shown in Fig. 3(b). Taking the square of the magnetization and extrapolating it linearly near the transition temperature to the intersection of a horizontal line from the minimum magnetization values, the Curie temperature was found to be approximately 535 K. A previous report on bulk iron carbide found a value of 483 K for the Fe_3_C phase.^6^ The value of 535 K is closer to what has been reported for Fe_7_C_3_ with a Curie temperature of 523 K (ref. 6) or Fe_5_C_2_ with a Curie temperature of 538 K.^30^ Tsuzuki *et al.* presented SEM images showing an irregular grain structure that is micrometres in size.^6^ In the case of Fe_5_C_2_ the Curie temperature value is given without indication of the microstructure.^30^ Since the as-synthesized sample is approximately 210 nm in size, it is unlikely that the difference in Curie temperature is attributed to a size effect when compared to the bulk. The difference in Curie temperatures may be related to our chemically synthesized Fe_3_C phase being more Fe rich relative to the reported bulk value by Tsuzuki *et al.* This is consistent with the lower saturation magnetization of 125 emu g^−1^ for their sample.^6^

## Conclusions

In conclusion, we developed a simple colloidal method for the synthesis of iron carbide nanoplatelets. The 1,2-hexadecanediol seems to play multiple roles in the reaction. First, it works as a reducing agent for Fe^3+^ reduction to the zero valence state, followed by a carbonization step at reaction temperatures higher than 300 °C. The sample synthesized at 320 °C exhibits a strong ferromagnetic behavior which is due to the presence of the iron carbide phase orthorhombic Fe_3_C. The sample had a high magnetization of 139 emu g^−1^ under a 30 kOe applied field. The simple solution synthesis method presented is promising for utilizing iron carbide nanomaterials for biomedical and catalytic applications.

## Conflicts of interest

There are no conflicts to declare.

## Supplementary Material

NA-001-C9NA00526A-s001

NA-001-C9NA00526A-s002

NA-001-C9NA00526A-s003
