# Supplementary figures and images for: Iron carbide nanoplatelets: colloidal synthesis and characterization
Source: Nanoscale Adv. 2019 Oct 7;1(11):4476–80. doi: 10.1039/c9na00526a (PMC9417806; doi:10.1039/c9na00526a)

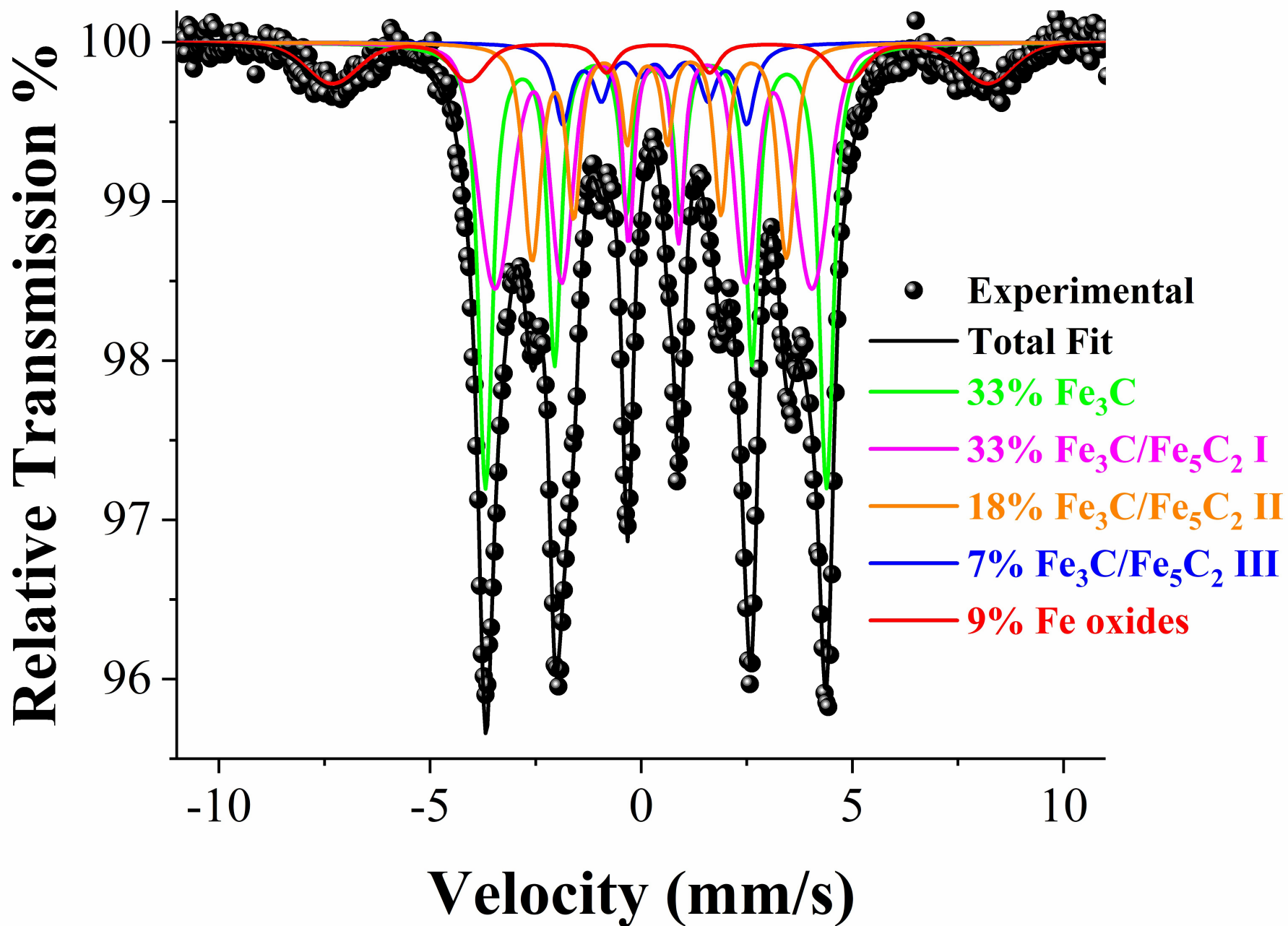

Supplement: NA-001-C9NA00526A-s002 [file NA-001-C9NA00526A-s002.pdf]
