# Supplementary material for: Iron carbide nanoplatelets: colloidal synthesis and characterization
Source: Nanoscale Adv. 2019 Oct 7;1(11):4476–80. doi: 10.1039/c9na00526a (PMC9417806; doi:10.1039/c9na00526a)
Supplement: NA-001-C9NA00526A-s003 [file NA-001-C9NA00526A-s003.pdf]

Please do not adjust margins

Supporting Information

Iron Carbide Nanoplatelet: Colloidal Synthesis and Characterization

Frank M. Abel,*[a] Shirin Pourmiri [a], Georgia Basina[b], Vasileios Tzitzios[*,b,c], Eamonn, Devlin[c], and George C. Hadjipanayis [a]

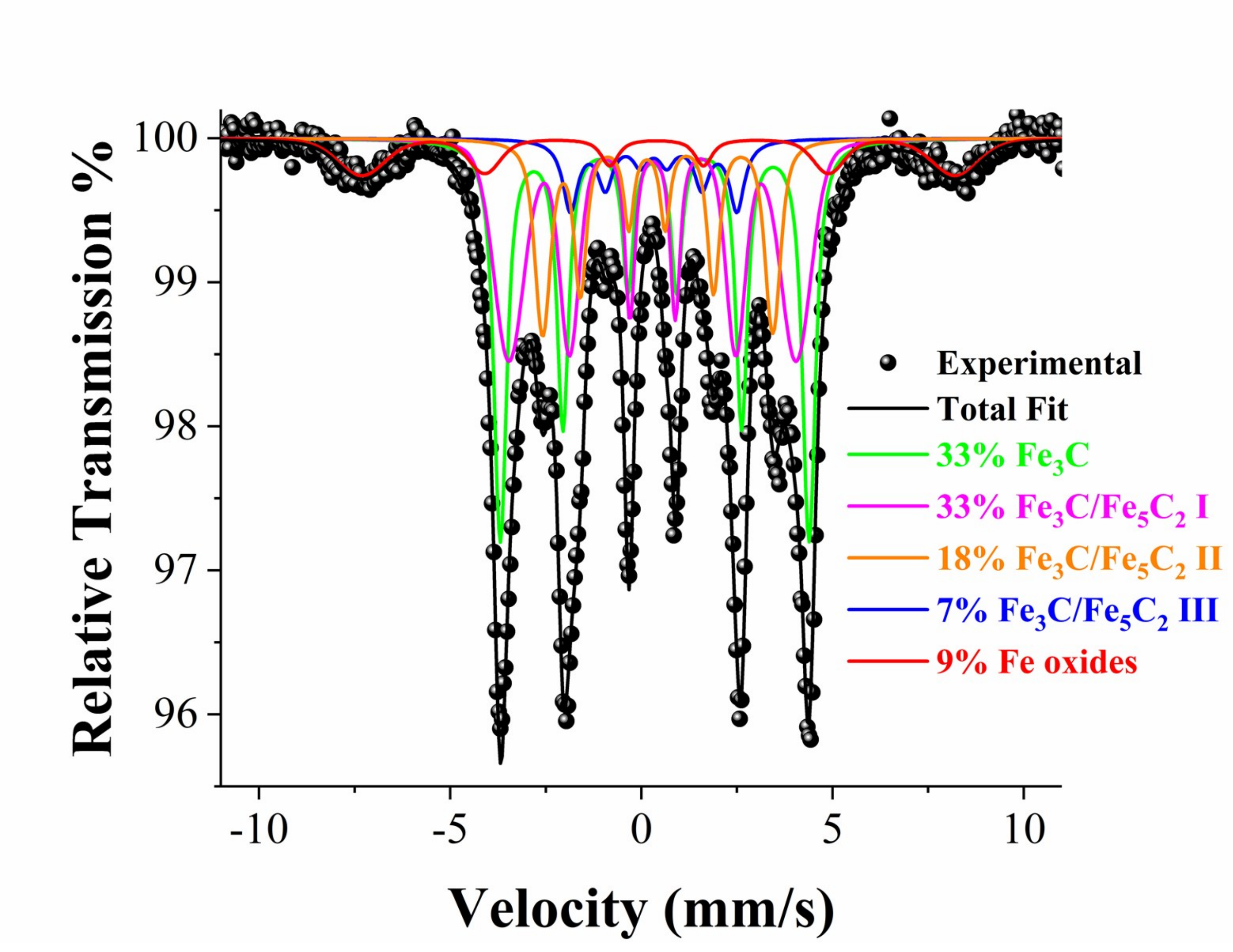

Figure S1. Mössbauer spectroscopy performed at 4.2 K of sample synthesized at 300 °C for 1 hour followed 340 °C for 2 hours, showing a mixture of iron carbide phases and a small percentage of iron oxides.

[a.] *Department of Physics and Astronomy, University of Delaware, Newark, DE 19716, USA.*
[b.] *Department of Chemical Engineering, Khalifa University of Science and Technology, Petroleum Institute, P.O. Box 2533, Abu Dhabi, United Arab Emirates.*
[c.] *Institute of Nanoscience and Nanotechnology, NCSR Demokritos, Athens, 15310, Greece*
** Corresponding Authors: Frank M. Abel, E-mail: fabel@udel.edu, frank.m.abeliii@gmail.com, Vasileios Tzitzios, E-mail: v.tzitzios@inn.demokritos.gr*

Please do not adjust margins
